# Tumor immune microenvironment in non-small cell lung cancer progression

**DOI:** 10.3389/fimmu.2026.1778251

**Published:** 2026-05-18

**Authors:** Jie Jiang, Mingqiang Zhong, Jian Wang, Zhiya Zhang, Wenqiang Li, Zhiping Deng

**Affiliations:** 1 Department of Respiratory and Critical Care Medicine, Zigong First People’s Hospital, Zigong, China; 2 Department of Oncology, Jiangjin Hospital, School of Medicine, Chongqing University, Chongqing, China; 3 Institute of Tuberculosis and Thoracic Tumors, Beijing Chest Hospital, Beijing, China

**Keywords:** adaptive immunity, B cell, immunotherapy, macrophage polarization, NSCLC, T cell exhaustion, tumor immune microenvironment

## Abstract

Non-small cell lung cancer (NSCLC), the most prevalent form of lung malignancy, remains a leading cause of cancer-related mortality despite advances in surgery, targeted therapy, and immunotherapy. A growing body of evidence implicates the tumor immune microenvironment (TIME) as a critical determinant of disease progression, immune evasion, and therapeutic resistance. This review synthesizes current insights into the roles of innate and adaptive immune populations—including tumor-associated macrophages, neutrophils, dendritic cells, mast cells, myeloid-derived suppressor cells, natural killer cells, T cells, and B cells—in shaping tumor dynamics. Special emphasis is placed on the phenotypic plasticity and functional reprogramming of these immune subsets within the TIME. We further discuss the implications of immune cell heterogeneity for immunotherapy response and resistance. By highlighting the interplay between tumor cells and the immune landscape, this review underscores the potential for TIME-informed strategies to optimize patient stratification and advance combinatorial immunotherapeutic approaches in NSCLC.

## 1 Introduction

Non-small cell lung cancer (NSCLC), which represents the majority of lung cancer cases, is characterized by an unfavorable five−year survival outcome (1). The emergence of molecularly targeted agents and immune checkpoint inhibitors (ICIs) has reshaped therapeutic paradigms in NSCLC; nevertheless, clinical responses to immunotherapy are observed in only about one−third of patients (2, 3). This constrained benefit is increasingly linked to the intricate tumor immune microenvironment (TIME), a multifaceted system that regulates malignant progression, immune escape, and resistance to therapy. The TIME comprises diverse cellular and non−cellular elements, including malignant cells, infiltrating immune populations, stromal and endothelial cells, as well as extracellular matrix structures, collectively forming a permissive milieu for tumor growth and dissemination (4).

Within the TIME, immune cells play a pivotal role under homeostatic conditions by identifying and eliminating aberrant or pathogen-infected cells. Yet, during tumor progression and immunoediting, these cells can be reprogrammed phenotypically, shifting toward either tumor-suppressive or tumor-supportive roles, shaped by the local milieu (5, 6). Tumor-derived factors—including cytokines, chemokines, and metabolic products—dynamically reshape immune responses, while immune cells, in turn, influence tumor evolution through soluble mediators and direct contact. A detailed comprehension of this reciprocal interaction is critical for pinpointing immune-based therapeutic opportunities in lung cancer (7, 8). Importantly, how does the dynamic state and plasticity of TIME may impact NSCLC progression and enable the development of more effective immunotherapies? To answer this question, this review summarizes emerging insights into immune subset characterization, tumor-immune interplay, and their relevance to refining immunotherapeutic interventions in NSCLC.

## 2 Innate immune cells

### 2.1 Macrophages

As central components of innate immunity, macrophages participate in the immediate defense against pathogens alongside other innate immune effectors, including neutrophils, dendritic cells (DCs), mast cells, myeloid-derived suppressor cells (MDSCs), and natural killer (NK) cells, all of which coordinate pathogen recognition and clearance (9). When recruited into the tumor milieu, these macrophages are termed tumor-associated macrophages (TAMs) and are critically involved in promoting metastasis, angiogenesis, immune evasion, and therapeutic resistance (10, 11). The remarkable plasticity of TAMs enables their polarization into distinct functional states in response to environmental cues within the tumor immune microenvironment (TIME) (12). TAM phenotypes in NSCLC exist along a dynamic continuum rather than within a strict binary framework. During lung cancer progression, a gradual predominance of M2-like macrophages over M1-like populations has been frequently observed (13, 14). M1-type macrophages enhance antitumor immunity by secreting inflammatory mediators such as TNF-α, IL-12, IL-1β, and reactive nitrogen intermediates, thereby recruiting and activating cytotoxic T cells and NK cells. In contrast, M2-polarized macrophages facilitate immune escape through the production of IL-10, TGF-β, VEGF, CCL17, and CCL22, which suppress effector lymphocyte responses, promote regulatory T-cell recruitment, and stimulate angiogenesis and extracellular matrix remodeling (15, 16).

Importantly, the functional reprogramming of TAMs is strongly driven by metabolic and cytokine-mediated pressures within the NSCLC TIME. Tumor hypoxia stabilizes HIF-1α signaling in macrophages, enhancing VEGF expression and favoring pro-angiogenic phenotypes. In parallel, lactate released by glycolytically active tumor cells promotes M2-like polarization through GPR81-dependent signaling and HIF-1α activation, while simultaneously suppressing pro-inflammatory cytokine production (17, 18). Lipid accumulation and oxidized fatty acids may further reinforce immunosuppressive macrophage states through PPARγ and FAO-associated metabolic pathways (19, 20). Tumor-derived CSF-1 and IL-34 sustain macrophage recruitment, survival, and differentiation through CSF1R signaling, whereas IL-10 and TGF-β activate STAT3 and SMAD2/3 pathways, inhibiting antigen presentation and inflammatory activation. Conversely, IFN-γ, GM-CSF, and Toll-like receptor ligands activate NF-κB and STAT1 signaling, promoting M1-like immune-stimulatory programs (21, 22). These interactions impair CD8^+^ T-cell cytotoxicity, reduce dendritic-cell maturation, and enhance vascular remodeling (11, 23). Notably, M2 macrophages accumulation has been observed in the stromal compartment of NSCLC (24, 25). Consequently, targeting TAMs has emerged as a promising strategy to enhance current anticancer therapies.

### 2.2 Neutrophils

Neutrophils serve as pivotal components of the innate immune system and are rapidly mobilized during acute inflammation to sites of tissue injury or infection in response to chemotactic gradients, where they eliminate pathogens through phagocytosis, degranulation, and the release of antimicrobial mediators (26, 27). Within TIME, infiltrating neutrophils are commonly termed tumor-associated neutrophils (TANs), which display remarkable phenotypic and functional plasticity. Depending on contextual cues, TANs may exert either anti-tumor or pro-tumor activities, thereby becoming critical regulators of NSCLC progression (28, 29). The polarization of TANs into anti-tumor N1-like or pro-tumor N2-like phenotypes is strongly regulated by cytokines and metabolic signals within the TIME. TGF-β is a major driver of N2 polarization, promoting arginase-1 expression, immunosuppressive mediator release, and NET formation. By contrast, type I interferons, particularly IFN-β, favor N1-like differentiation characterized by enhanced cytotoxicity and inflammatory cytokine production (30, 31). Chemokines such as (IL-8, CXCL1, and CXCL5, acting through CXCR1/CXCR2 signaling, are major mediators of neutrophil recruitment into NSCLC lesions. Tumor hypoxia and granulocyte-macrophage colony-stimulating factor (GM-CSF) prolong neutrophil survival via PI3K/AKT and ERK signaling pathways, whereas lactate-rich acidic environments promote suppressive neutrophil phenotypes and impair anti-tumor immunity (32, 33). TANs also contribute to tumor suppression by engaging Fc receptors to detect tumor cells and releasing cytotoxic mediators including ROS and MPO, thereby triggering antibody-dependent cellular cytotoxicity (ADCC) (34, 35). In contrast, TANs also promote malignancy through the secretion of proteolytic enzymes such as neutrophil elastase (NE) and matrix metalloproteinases (MMPs), which enhance neovascularization and support cancer cell invasion and dissemination (36). Therapeutically, TANs are emerging as attractive targets in NSCLC. Strategies aimed at inhibiting CXCR2-mediated recruitment, blocking TGF-β signaling, reducing NETosis, or reprogramming TANs toward an N1-like phenotype may enhance responsiveness to immune checkpoint blockade and conventional therapies (34, 37). Nonetheless, the molecular determinants governing neutrophil heterogeneity, lineage plasticity, and treatment-induced adaptation remain incompletely understood and warrant further investigation.

### 2.3 Dendritic cells

DCs, the most competent professional antigen-presenting cells (APCs), are broadly distributed throughout the circulatory system, secondary lymphoid structures, and peripheral tissues (38). In the context of tumors, DCs infiltrating the TIME are termed tumor-associated dendritic cells (TADCs). Their principal role is to detect and internalize tumor-associated antigens (TAAs), process them into peptides, and present them on MHC molecules to naïve T cells, initiating cytotoxic responses against malignant cells. This immunological priming depends on enhanced expression of co-stimulatory ligands and secretion of immunostimulatory chemokines (39, 40). While immature DCs exhibit strong migratory behavior, mature DCs possess robust capacity to stimulate naïve T cells and coordinate adaptive immunity (39, 41). However, the scarcity or functional impairment of mature DCs within tumors constitutes a significant limitation to effective immunotherapeutic outcomes. In a non-immunogenic state, DCs exhibit poor antigen presentation, thus failing to support the activation of tumor-specific T cells and permitting immune escape (42). Lung carcinoma cells can release suppressive cytokines such as interleukin (IL)-10 and IL-6, which inhibit DC maturation, skew differentiation, and disrupt phenotypic programming—thereby impairing their immunostimulatory capacity and facilitating tumor advancement (43). Clinical observations indicate a positive relationship between DC abundance and survival in lung adenocarcinoma. Tumors with fewer mature TADCs and a predominance of immature DCs are generally associated with greater malignancy (44, 45). Dendritic cell–based vaccines, employing ex vivo–cultured autologous DCs pulsed with tumor antigens, have shown promise by reactivating cytotoxic T lymphocytes (CTLs) upon reinfusion (46, 47). These therapeutic platforms are being increasingly integrated into treatment strategies for lung cancer. Continued exploration of the dynamic crosstalk between DCs and tumor cells remains essential for advancing immunotherapeutic precision.

### 2.4 Mast cells

Mast cells, integral to innate immunity, are predominantly located adjacent to microvessels within the dermis and visceral submucosa. During early tumorigenesis, they represent one of the initial immune cell types to accumulate at the tumor margins (48, 49). Recent findings suggest that mast cells exhibit paradoxical roles in cancer, functioning both as tumor promoters and suppressors. Pro-tumorigenic activities include the enhancement of angiogenesis, release of autocrine mediators, and secretion of diverse growth-stimulatory molecules. Conversely, mast cells may hinder tumor advancement by discharging proteolytic enzymes upon degranulation, which degrade the extracellular matrix and compromise tumor cell integrity (50, 51). Notably, intratumoral mast cells often display an activated state, and their numbers tend to escalate with disease progression (52). Thus, evaluating mast cell infiltration within the TIME holds potential for early detection and prognostic assessment in lung carcinoma.

### 2.5 Myeloid-derived suppressor cells

MDSCs comprise a diverse group of immunosuppressive cells, primarily arising from immature myeloid precursors such as monocytes, granulocytes, and dendritic cell progenitors (53). In disease states, the normal trajectory of myeloid differentiation becomes dysregulated, resulting in aberrant expansion and systemic accumulation of MDSCs (54, 55). Within the lung cancer microenvironment, these cells exert potent immunosuppressive effects by secreting a repertoire of inhibitory molecules that compromise the function of cytotoxic T lymphocytes (CTLs), NK cells, and antigen-presenting dendritic cells, collectively dampening anti-tumor immunity (56). Moreover, MDSCs facilitate tumor advancement by releasing pro-angiogenic mediators that promote neovascularization and support immune escape, thereby contributing to tumor proliferation, invasion, and metastatic dissemination (57, 58). Tumor progression itself drives MDSC recruitment, reinforcing immunosuppression and establishing a vicious cycle of malignancy. *In vitro* analyses have shown that MDSCs derived from lung squamous cell carcinoma induce apoptosis in CD8^+^ T cells, impairing their cytolytic capacity (59, 60); a similar mechanism has been reported in lung adenocarcinoma. Notably, increased MDSC burden is associated with poorer prognosis in patients with lung cancer (61). Clinical interventions—including surgery, radiotherapy, and chemotherapy—have been observed to diminish MDSC levels, promote their differentiation, curb their expansion, and limit their infiltration into tumor niches. Therefore, therapeutic strategies aimed at curbing MDSC accumulation or suppressive function are of significant interest (62). Nevertheless, elucidating the precise molecular pathways that control MDSC ontogeny and activity remains critical for identifying effective and selective therapeutic targets.

### 2.6 Natural killer cells

**Figure 1:**
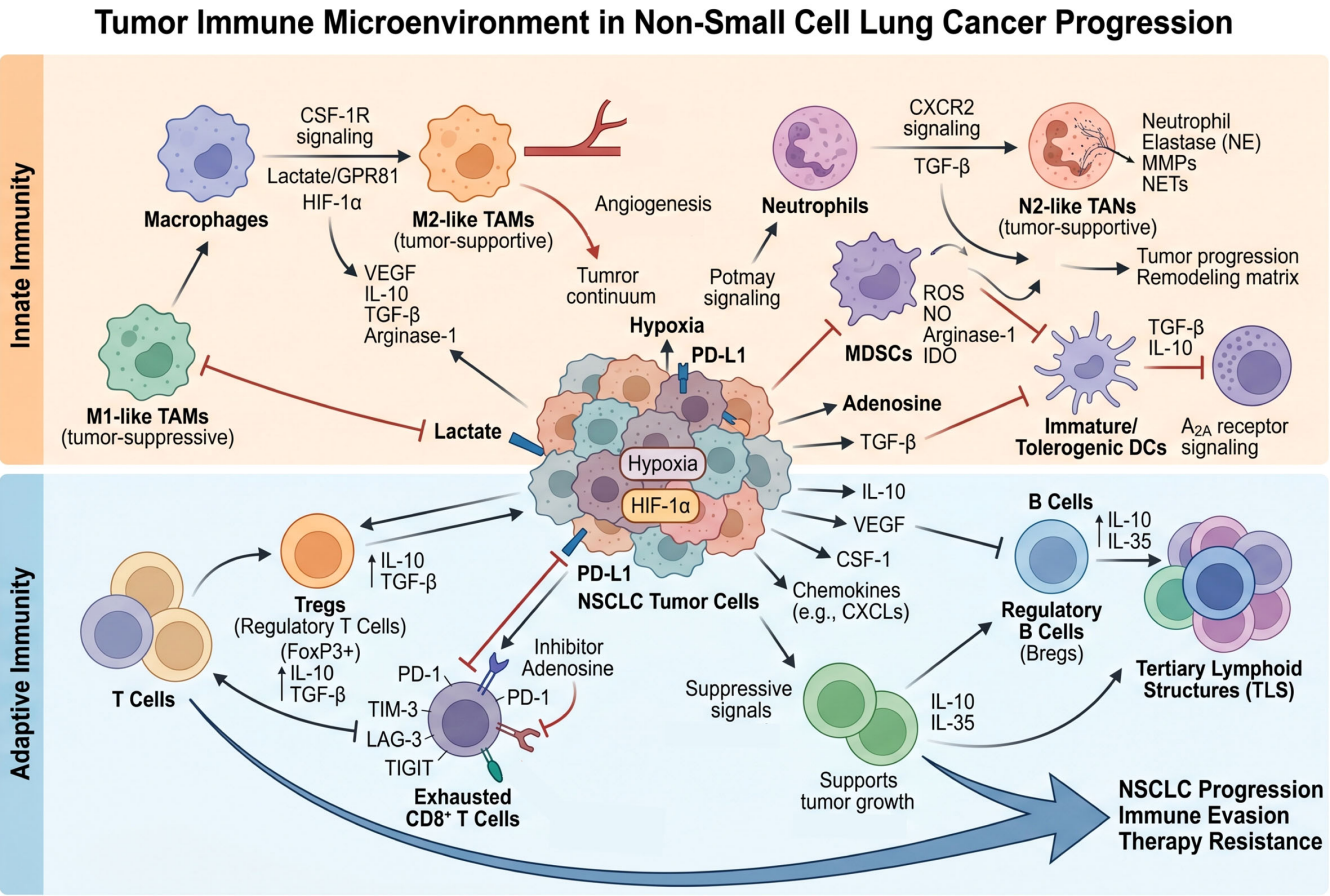
Immune cells in non-small cell lung cancer progression.

NK cells are pivotal components of innate immunity, functioning at the interface of innate and adaptive responses (63, 64). In lung cancer, however, NK-cell efficacy is frequently compromised by the profoundly suppressive conditions of the TIME. In NSCLC, NK-cell density is significantly reduced and exhibits marked spatial heterogeneity, with relatively higher abundance in stromal compartments but limited penetration into the tumor core (65). This exclusion pattern suggests that both physical stromal barriers and inhibitory biochemical cues restrict NK-cell trafficking and persistence within malignant nests. Tumor-derived TGF-β is a central suppressor of NK-cell activity, as it downregulates activating receptors such as NKG2D and NKp30, inhibits perforin and granzyme synthesis, and promotes a less cytotoxic phenotype. In parallel, IL-10 and prostaglandin E2 further attenuate NK-cell activation and cytokine secretion. Metabolic constraints also critically contribute to NK-cell dysfunction in NSCLC (66, 67). Hypoxia stabilizes HIF-1α signaling and impairs mitochondrial fitness, thereby reducing NK-cell proliferation and persistence (68). Excess extracellular lactate generated by glycolytic tumor cells acidifies the microenvironment and suppresses IFN-γ production, whereas adenosine produced through CD39/CD73 ectonucleotidase pathways activates A2A receptors to inhibit cytotoxic degranulation (69, 70). Chronic exposure to PD-L1^high^ tumor cells and suppressive myeloid populations may additionally induce an exhausted NK-cell phenotype characterized by upregulation of inhibitory receptors such as TIGIT, TIM-3, NKG2A, and PD-1, further limiting anti-tumor surveillance (71, 72). Despite these barriers, NK cells remain attractive therapeutic effectors in lung malignancies. Adoptive NK-cell therapy entails the isolation of autologous or allogeneic NK cells, followed by ex vivo activation and expansion prior to reinfusion, enabling direct cytotoxicity against tumor cells and secondary stimulation of adaptive immunity (73). Pharmacologic strategies—including IL-15 superagonists, checkpoint blockade targeting NKG2A or TIGIT, STING agonists, and monoclonal antibodies designed to enhance ADCC—can reinvigorate NK-cell function and improve tumor clearance (74, 75). Emerging approaches such as chimeric antigen receptor NK (CAR-NK) cells and combination regimens integrating NK-cell therapy with PD-1/PD-L1 inhibitors may further overcome immune resistance in NSCLC (76, 77). Therefore, restoring NK-cell infiltration, metabolic fitness, and effector competency represents a promising avenue for precision immunotherapy in lung cancer (**Figure 1**).

## 3 Adaptive immune cells

### 3.1 T cells

Tumor-infiltrating lymphocytes (TILs), particularly CD4^+^ and CD8^+^ subsets, constitute a pivotal element in cancer immunosurveillance (78, 79). CD4^+^ T cells, which function as helper subsets (Th), coordinate immune regulation via cytokine secretion (80). Within the TIME, these cells differentiate into Th1, Th2, Th17, and Tregs (81). Th1 cells exert antitumor functions by releasing IL-2, IFN-γ, and TNF, thereby promoting apoptosis and impeding angiogenesis. In contrast, Th2 cells secrete IL-4, IL-5, IL-13, and IL-10, favoring immunomodulatory activities (82). Notably, a shift within the TIME—marked by declining Th1 cells and rising Th2 populations—leads to a diminished Th1/Th2 ratio. Th17 cells enhance antitumor defense by attracting neutrophils and triggering inflammatory mediator release, thereby restraining tumor growth (81, 83). Tregs, by contrast, dampen immune activation through IL-4, IL-10, and TGF-β, fostering immune evasion and facilitating tumor expansion (84). The suppressive functions of Tregs are well-characterized in lung cancer, identifying them as promising targets for immunotherapeutic strategies. CD8^+^ T lymphocytes act predominantly as cytotoxic effectors, directly eliminating tumor cells and constituting a critical arm of anti-cancer immunity (85, 86). Nevertheless, the upregulation of CTLA-4 and PD-1 can impair their function, diminish CD4^+^ T cell–mediated tumor surveillance, and facilitate tumor progression (87, 88). In NSCLC, there is a notable reduction in CD4^+^ and CD8^+^ T cell infiltration within tumor tissues, accompanied by a substantially increased CD4^+^/CD8^+^ ratio (79). Both the abundance of infiltrating CD8^+^ T cells and the ratio between CD4^+^ and CD8^+^ subsets exhibit strong prognostic value in NSCLC patients (89).

In addition to subset composition, the spatial arrangement of T cells is a crucial determinant of T cell efficacy in NSCLC. Tumors enriched with parenchymal CD8^+^ T cell infiltration generally exhibit a more inflamed phenotype and greater sensitivity to immune checkpoint blockade, whereas tumors in which T cells remain confined to the stromal compartment or invasive margin often display immune exclusion and reduced therapeutic responsiveness (90, 91). This spatial segregation is regulated by multiple mechanisms, including TGF-β-driven fibroblast activation, CXCL12/CXCR4-mediated retention, abnormal vascular signaling, and extracellular matrix stiffening, all of which impede T-cell entry into tumor nests (92, 93). Once excluded, T cells are further exposed to suppressive mediators such as IL-10, adenosine, and VEGF, as well as chronic antigen stimulation that promotes the upregulation of inhibitory receptors including PD-1, TIM-3, LAG-3, and TIGIT (94, 95). At the signaling level, these processes are associated with activation of SMAD2/3, STAT3, PI3K/AKT, and NFAT-related exhaustion programs, ultimately weakening cytotoxicity and cytokine production (96). Therefore, evaluating whether T cells are present merely at the tumor periphery or are capable of deep parenchymal infiltration is essential for understanding functional immune competence in NSCLC.

### 3.2 B cells

B lymphocytes arise through differentiation from hematopoietic stem cells and become functionally engaged following antigen exposure, a process that culminates in germinal center development (97–99). B cell infiltration is markedly enriched in lung cancer specimens relative to peritumoral or distant normal tissues, with this phenomenon being particularly pronounced in NSCLC (100, 101). Functionally, tumor−infiltrating B cells exert context−dependent immunoregulatory effects during cancer evolution. At early disease stages, B cells predominantly display tumor−restraining activity through multiple pathways, including antibody−mediated ADCC, antigen presentation that facilitates CD4^+^ T−cell priming, and the enhancement of CD8^+^ T−cell activation and clonal expansion, collectively limiting malignant progression (102). Regulatory B cells (Bregs), a distinct subset of tumor-infiltrating B lymphocytes, exert potent immunoregulatory and suppressive functions. They release immunosuppressive cytokines such as IL-10, IL-35, and TGF-β, which collectively suppress anti-tumor immune responses (103, 104). Tumor-driven Bregs contribute to immune escape by shielding malignant cells from cytotoxic immune surveillance. Moreover, in densely populated tumor niches, direct Breg–tumor interactions have been shown to enhance NSCLC progression (66, 105). Spatially, B cells in NSCLC are often enriched within stromal immune aggregates and tertiary lymphoid structures (TLSs) (106, 107). This organization has important functional implications. B cells localized in TLS-like regions may support antitumor immunity by promoting antigen presentation, plasma-cell differentiation, and T-cell activation, whereas B cells skewed toward a regulatory phenotype in suppressive stromal regions may instead reinforce immune tolerance (108, 109). Cytokines such as IL-21, CXCL13, BAFF, IL-10, and TGF-β, together with signaling pathways including STAT3, NF-κB, and B-cell receptor–associated cascades, shape B-cell differentiation and function within these spatially distinct niches (110, 111). Accordingly, the prognostic value of B-cell infiltration in NSCLC likely depends not only on quantity, but also on whether B cells are organized within immune-supportive TLS structures or embedded within suppressive stromal microdomains.

## 4 Conclusion

The immunological landscape of NSCLC is characterized by a dynamic and heterogeneous TIME, where immune cells can adopt both pro- and anti-tumor phenotypes depending on local cues. Innate immune components such as TAMs, TANs, and MDSCs often dominate the immunosuppressive niche, promoting tumor growth, angiogenesis, and resistance to therapy. Conversely, dendritic cells and NK cells exhibit potent antitumor activity but are frequently functionally impaired. Adaptive immune subsets, including CD4^+^ and CD8^+^ T cells and B cells, display diverse roles ranging from immune surveillance to immunosuppression, especially when regulatory phenotypes prevail. The shifting balance of these immune populations determines the trajectory of tumor evolution and the success of immune-based treatments.

Moving forward, a major priority in NSCLC research will be to translate TIME heterogeneity into clinically actionable stratification frameworks. Rather than treating the TIME as a uniform entity, patients may be more precisely categorized into immune-inflamed, immune-excluded, or immune-desert phenotypes, as well as into myeloid-enriched, T-cell-exhausted, or TLS-supportive immune contexts. Such classification may improve prediction of therapeutic responsiveness and support more rational treatment allocation. For example, immune-inflamed tumors with abundant intratumoral CD8^+^ T-cell infiltration may be more likely to benefit from immune checkpoint blockade, whereas immune-excluded tumors may require stromal remodeling or anti-TGF-β strategies to restore lymphocyte penetration. In parallel, myeloid-dominant TIME subtypes characterized by TAM, TAN, or MDSC accumulation may be particularly suitable for combination regimens integrating ICIs with CSF1R inhibition, CXCR2 blockade, anti-angiogenic therapy, or other myeloid-reprogramming strategies. Accordingly, future therapeutic design should increasingly emphasize biomarker-guided combination approaches that match distinct TIME states with tailored interventions, including checkpoint inhibition, stromal normalization, metabolic reprogramming, cytokine/chemokine-axis targeting, and adoptive cellular immunotherapy. The integration of spatial transcriptomics, multiplex imaging, and single-cell profiling into clinical decision-making may further refine this stratification paradigm and accelerate the development of precision immuno-oncology for NSCLC.
